# Human Catestatin Alters Gut Microbiota Composition in Mice

**DOI:** 10.3389/fmicb.2016.02151

**Published:** 2017-01-17

**Authors:** Mohammad F. Rabbi, Peris M. Munyaka, Nour Eissa, Marie-Hélène Metz-Boutigue, Ehsan Khafipour, Jean Eric Ghia

**Affiliations:** ^1^Department of Immunology, University of ManitobaWinnipeg, MB, Canada; ^2^Department of Animal Sciences, University of ManitobaWinnipeg, MB, Canada; ^3^Biomaterials and Tissue Engineering, Institut National de la Santé et de la Recherche MédicaleStrasbourg, France; ^4^Department of Medical Microbiology, University of ManitobaWinnipeg, MB, Canada; ^5^The Children's Hospital Research Institute of ManitobaWinnipeg, MB, Canada; ^6^Section of Gastroenterology, Department of Internal Medicine, University of ManitobaWinnipeg, MB, Canada; ^7^Inflammatory Bowel Disease Clinical and Research Centre, University of ManitobaWinnipeg, MB, Canada

**Keywords:** gut microbiota, intestinal homeostasis, chromogranin A (CgA), catestatin (CST), antimicrobial peptides, microbial dysbiosis

## Abstract

The mammalian intestinal tract is heavily colonized with a dense, complex, and diversified microbial populations. In healthy individuals, an array of epithelial antimicrobial agents is secreted in the gut to aid intestinal homeostasis. Enterochromaffin cells (EC) in the intestinal epithelium are a major source of chromogranin A (CgA), which is a pro-hormone and can be cleaved into many bioactive peptides that include catestatin (CST). This study was carried out to evaluate the possible impact of CST on gut microbiota *in vivo* using a mouse model. The CST (Human CgA_352−372_) or normal saline was intrarectally administered in C57BL/6 male mice for 6 days and then sacrificed. Feces and colonic mucosa tissue samples were collected, DNA was extracted, the V4 region of bacterial 16S rRNA gene was amplified and subjected to MiSeq Illumina sequencing. The α-diversity was calculated using Chao 1 and β-diversity was determined using QIIME. Differences at the genus level were determined using partial least square discriminant analysis (PLS-DA). Phylogenetic investigation of communities by reconstruction of unobserved states (PICRUSt) was used to predict functional capacity of bacterial community. CST treatment did not modify bacterial richness in fecal and colonic mucosa-associated microbiota; however, treatment significantly modified bacterial community composition between the groups. Also, CST-treated mice had a significantly lower relative abundance of Firmicutes and higher abundance of Bacteroidetes, observed only in fecal samples. However, at lower phylogenetic levels, PLS-DA analysis revealed that some bacterial taxa were significantly associated with the CST-treated mice in both fecal and colonic mucosa samples. In addition, differences in predicted microbial functional pathways in both fecal and colonic mucosa samples were detected. The results support the hypothesis that CST treatment modulates gut microbiota composition under non-pathophysiological conditions, however, the result of this study needs to be further validated in a larger experiment. The data may open new avenues for the development of a potential new line of antimicrobial peptides and their use as therapeutic agents to treat several inflammatory conditions of the gastrointestinal tract, such as inflammatory bowel disease (IBD), inflammatory bowel syndrome (IBS), or other health conditions.

## Introduction

Over the last 15 years, bacterial multi-drug resistance (MDR) has emerged as a result of several socio-economical reasons, such as the use of surface antibacterial agents that are now available in many household products (Davies and Davies, 2010), antibiotic over-prescription, or failing to complete a course of antibiotics (Davies and Davies, 2010). Although due to MDR new line of antibiotics are required, development of new antibiotics has been reduced by pharmaceutical companies because of the cost and complexity of clinical trials (Mullard, 2014). Currently, there are relatively few new antimicrobials in development.

The gastrointestinal (GI) tract is heavily colonized with an average of 10^14^ microbes that represent thousands of species, which is 10 times more than the total number of cells in the human body (Yu and Huang, 2013). More than 90% of members of this bacterial community belong to two major phyla: gram-negative Bacteriodetes and gram-positive Firmicutes (Peterson et al., 2008; Kaser et al., 2010), with the remaining belong to low-abundance phyla such as Proteobacteria and Actinobacteria. Viruses, protists, and fungi are also other members of gut microbiome (Peterson et al., 2008; Kaser et al., 2010). In healthy individuals and during certain age categories, microbial diversity in the intestine is stable over time and demonstrates a symbiotic relationship with the host (Yu and Huang, 2013), but a shift in microbial composition, referred to as dysbiosis, has been described in several pathologies (Kallus and Brandt, 2012; Collins, 2014; Carding et al., 2015). Gut microbiota helps to digest food items. Various metabolites produced by the resident microbiota play a significant role in host physiology, metabolism and immune function. For example, gut microbiota can activate toll-like receptors (TLRs) in the gut epithelium, which in turn can affect the expression of antimicrobial peptides, such as angiogenins (Vaishnava et al., 2008; Raybould, 2012). In addition to the innate immune system, gut microbiota can also control the host's adaptive immune system through T cell receptor αβ-positive intraepithelial lymphocytes, regulatory T cells and T helper 17 cells (Kaser et al., 2010). Overall, gut homeostasis is largely dependent on the proper balance and composition of gut microbiome (Stecher and Hardt, 2008).

At the mucosal level the epithelium plays a major role in limiting the passage/penetration of bacteria to the sub-mucosa from the gut lumen. Antimicrobial peptides (AMPs) secreted by epithelial cells have a broad spectrum effect against bacteria and are a part of an ancient defense mechanism that is present in virtually all mammals (Ostaff et al., 2013). In the GI tract, specialized intestinal epithelial cells or circulating inflammatory cells are a major source of these AMPs (Ostaff et al., 2013). Within the epithelium, Paneth cells are the main producer of AMPs but new data indicate that enterochromaffin (EC) cells can hypothetically also produce certain types of AMPs (Khan and Ghia, 2010).

EC cells are the major source of chromogranin A (CgA; Norlén et al., 2001), a family of highly acidic proteins. The CgA gene is localized at 14q32 in the human genome, consisting of eight exons and seven introns, and its 2-Kb transcript is translated into the 457-residue CgA protein. The overall homology for CgA in different vertebrates is ~40%, but the most highly conserved regions occur at the N- and C-termini, which show up to 88% sequence homology. Cell- and tissue-specific CgA processing has been described in the rat, mouse, and human GI tract (Curry et al., 1991; Portela-Gomes and Stridsberg, 2001, 2002). The CgA primary structure from its cDNA sequence shows the presence of numerous pairs of basic amino acids. These are potential sites for cleavage by prohormone convertases (PC) 1/3 or 2, and carboxypeptidase E/H (Seidah and Chrétien, 1999), which is consistent with evidence that CgA may serve as a prohormone for shorter bioactive fragments (Eiden, 1987); this is also suggested by the high sequence conservation of CgA-derived peptides. But in the gut, peptides can be highly sensitive to enzymes present in the luminal environment. Proteolytic fragments of CgA-derived peptides exert a broad spectrum of regulatory activities on the cardiovascular, endocrine and immune systems. Among its highly conserved C-terminal regions, CgA gives rise to a peptide of biological importance: the antihypertensive peptide catestatin (human CST; CgA_352−372_) (Mahata et al., 1997, 2010; Mahapatra et al., 2005), which has restricted antimicrobial activity against *Staphylococcus aureus in vitro* (Briolat et al., 2005). Similar to other AMPs, CST can interact with anionic components of fungi and bacteria. As a result, the microbial membrane is permeabilized, leading to cell lysis (Boman et al., 1993). *In vitro* studies have demonstrated that CST is effective against gram-positive bacteria, such as *S. aureus* and group A *Streptococcus*; gram-negative bacteria, such as *Escherichia coli, Pseudomonas aeruginosa*; yeasts, such as *Candida albicans*; and filamentous fungi, such as *Aspergillus niger, Aspergillus fumigatus*, and *Trichophyton rubrum* (Boman et al., 1993; Dorschner et al., 2001). However, to date, there has been no indication that the *in vitro* data can be reproduced using an *in vivo* model and whether or not the effect of CST would be similar in different gut compartment as the colonic mucosa-associated populations differ from the populations present in the feces (Zoetendal et al., 2002).

Despite the effects of CST on *S. aureus, E. coli*, and *P. aeruginosa* populations *in vitro*, the effects of *in vivo* CST treatment on microbiota across the GI tract is unknown. Our aim was to assess the compositional shifts and functional alterations in the fecal and colonic mucosa-associated microbiota in mice that were exposed to CST for 6 days.

## Materials and methods

### Animals

Male C57BL/6 mice (7–9 weeks old) were purchased from Charles River (Canada) and maintained in the animal care facility at the University of Manitoba. The experimental protocol was approved by the University of Manitoba Animal Ethics Committee (15-010) and the research was conducted according to the Canadian Guidelines for Animal Research (Gauthier, 2002; Demers et al., 2006). Two groups of four and eight mice were studied, one receiving the vehicle solution and one receiving intra-rectal (i.r.) infusion of CST for 6 days. By using mice from the same sex, source, age, and keeping them in co-housed conditions while receiving the same food, the environmental effects on gut microbiota were minimized.

### Peptide

The CST (Human CgA_352−372_: SSMKLSFRARAYGFRGPGPQL) (Mahata et al., 2010) was used (Biopeptide Co., Inc., San Diego, CA, USA), and the peptide was injected (i.r.) at 1.5 mg/per kg body weight per day for 6 days. Saline (0.9%) was injected in the control group. Mice were anesthetized using isoflurane (Abbott, Toronto, ON, Canada). PE-90 tubing (10 cm long; ClayAdam, Parisppany, NJ, USA), which was attached to a tuberculin syringe (BD, Mississauga, ON, Canada), was inserted 3.5 cm into the colon. The dose was determined according to our previous published study (Rabbi et al., 2014).

### Assessment of physiological condition

Weight loss, stool consistency, and bleeding were assessed daily to determine any possible physical changes in the mice as a result of CST treatment (Cooper et al., 1993). Scores were defined as follows: weight: 0, no loss; 1, 5–10%; 2, 10–15%; 3, 15–20%; and 4, 20% weight loss; stool: 0, normal; 2, loose stool; and 4, diarrhea; and bleeding: 0, no blood; 2, presence of blood; and 4, gross blood. Blood was assessed using the Hemoccult II test (Beckman Coulter, Oakville, ON, Canada).

### Fecal and tissue sample collection

Samples were collected 6 days post-treatment induction, after euthanasia under isoflurane (Abbot) anesthesia. The macroscopic score was determined on the sacrifice day based on stool consistency, rectal prolapse, and rectal and colonic bleeding. On the day of sacrifice, the colon was opened and approximately a 250 mg fecal sample was collected near the rectal opening. In addition, a portion of the colon tissue was collected within 5 cm from rectal opening. Approximately 50 mg of mucosa scrapings were collected from these colon tissue. All samples were collected in individual collector tubes from each animal and snap frozen in liquid nitrogen and preserved at −80°C until use.

### DNA extraction and quality check

Samples were homogenized at room temperature, and genomic DNA was extracted from mucosa scarpings using a ZR Tissue and Insect DNA extraction Kit (Zymo Research Corp., Orange, CA, USA). Fecal DNA extraction was performed using a ZR fecal DNA extraction kit (Zymo Research Corp., Orange, CA). Both DNA extraction kits had a bead-beating step to mechanically lyse microbial cells. DNA was quantified using a Nanodrop 2000 spectrophotometer (Thermo Scientific, Wilmington, DE, USA). DNA samples were normalized to achieve a concentration of 20 ng/μl, and quality-checked by PCR amplification of 16S rRNA gene using primers 27F (5′-GAAGAGTTTGATCATGGCTCAG-3′) and 342R (5′-CTGCTGCCTCCCGTAG-3′) (Sepehri et al., 2007; Khafipour et al., 2009). Amplicons were verified by agarose gel electrophoresis.

### Library construction and illumina sequencing

Library construction and Illumina sequencing were performed as described by Derakhshani et al. (2016) Briefly, the V4 region of the 16S rRNA gene was targeted for PCR amplification using modified F515/R806 primers (Caporaso et al., 2012). A reverse PCR primer was indexed with 12-base Golay barcodes to allow for sample multiplexing. The PCR reaction for each sample was performed in duplicate and contained 1.0 μl of pre-normalized DNA, 1.0 μl each of forward and reverse primers (10 μM), 12 μl HPLC grade water (Fisher Scientific, Ottawa, ON, Canada) and 10 μl 5 Prime Hot MasterMix (5 Prime, Inc., Gaithersburg, MD, USA). Reactions consisted of an initial denaturing step at 94°C for 3 min followed by 35 amplification cycles at 94°C for 45 s, 50°C for 60 s and 72°C for 90 s; this was followed by an extension step at 72°C for 10 min in an Eppendorf Mastercycler (Eppendorf, Hamburg, Germany). PCR products were then purified using a ZR-96 DNA Clean-up Kit (ZYMO Research, Irvine, CA, USA) to remove primers, dNTPs and reaction components. The V4 library was then generated by pooling 200 ng of each sample, and quantified using Picogreen dsDNA (Invitrogen, Carlsbad, CA, USA). This was followed by multiple dilution steps using pre-chilled hybridization buffer (HT1) (Illumina, San Diego, CA, USA) to bring the pooled amplicons to a final concentration of 5 pM, and the concentration was measured through optical density using a Qubit 2.0 Fluorometer (Life technologies, Burlington, ON, Canada). Finally, 15% of the PhiX control library was spiked into the amplicon pool to improve the unbalanced and biased base composition, a known characteristic of low diversity 16S rRNA libraries. Customized sequencing primers for read1 (5′-TATGGTAATTGTGTGCCAGCMGCCGCGGTAA-3′), read2 (5′-AGTCAGTCAGCCGGACTACHVGGGTWTCTAAT-3′), and index read (5′-ATTAGAWACCCBDGTAGTCCGGCTGACTGACT-3′) were synthesized and purified using polyacrylamide gel electrophoresis (Integrated DNA Technologies, Coralville, IA, USA) and added to the MiSeq Reagent Kit V2 (300-cycle) (Illumina, CA, USA). The 150 paired-end sequencing reaction was performed on a MiSeq platform (Illumina, CA, USA) at the Gut Microbiome and Large Animal Biosecurity Laboratories, Department of Animal Science, University of Manitoba, Canada. The sequencing data are uploaded into the Sequence Read Archive (SRA) of NCBI (http://www.ncbi.nlm.nih.gov/sra) and are accessible through accession number SRR2830596.

### Bioinformatics analyses

Bioinformatics analyses were performed as described previously (Derakhshani et al., 2016). Briefly, the PANDAseq assembler (Masella et al., 2012) was used to merge overlapping paired-end Illumina fastq files. All the sequences with mismatches or ambiguous calls in the overlapping region were discarded. The output fastq file was then analyzed using downstream computational pipelines in the open source software package QIIME (Caporaso et al., 2010a). Chimeric reads were filtered using UCHIME (Edgar et al., 2011) and sequences were assigned to operational taxonomic units (OTU) using the QIIME implementation of UCLUST (Edgar, 2010) at the 97% pairwise identity threshold. Taxonomies were assigned to the representative sequence of each OTU using an RDP classifier (Wang et al., 2007) and aligned with the Greengenes (v. 13.5) core reference database (DeSantis et al., 2006) using PyNAST algorithms (Caporaso et al., 2010b). The phylogenetic tree was built with FastTree 2.1.3 (Price et al., 2010) for additional comparisons between microbial communities.

### Alpha (α)- and beta (β)-diversity analyses

Within-community diversity (α-diversity) was calculated using QIIME and differences between control and CST groups were determined using SAS (SAS 9.3). An α rarefaction curve was generated using a Chao 1 estimator of species richness (Chao, 1984) with 10 sampling repetitions at each sampling depth. An even depth of ~15,700 sequences per sample was used for calculation of richness and diversity indices. To compare microbial composition between samples, β-diversity was measured by calculating the weighted and unweighted UniFrac distances (Lozupone and Knight, 2005) using QIIME default scripts. Principal coordinate analysis (PCoA) was applied on the resulting distance matrices to generate two-dimensional plots using PRIMER v6 software (Warwick and Clarke, 2006). Permutational multivariate analysis of variance of Bray-Curtis distance (PERMANOVA; Anderson, 2005) was used to calculate *P*-values and test for significant differences in β-diversity among treatment groups.

### Partial least square discriminant analysis

Partial least square discriminant analysis (PLS-DA; SIMCA P+ 13.0, Umetrics, Umea, Sweden) was performed on the genus data to identify the effects of treatments (Li et al., 2012; Derakhshani et al., 2016). The PLS-DA is a particular case of partial least square regression analysis in which Y is a set of variables describing categories of variables on X. In this case, X variables were the bacterial genera and the Y variables were observations of different treatment groups compared together. To avoid over-parameterization of the model, the variable influence on the projection (VIP) value was estimated for each genus, and genera with VIP < 0.50 were removed from the final model (Pérez-Enciso and Tenenhaus, 2003; Verhulst et al., 2011). *R*^2^ estimate then was used to evaluate the goodness of fit and *Q*^2^ estimate was used to evaluate the predictive value of the model. The PLS-regression coefficients were used to identify genera that were most characteristic of each treatment group and the results were visualized by PLS-DA loading scatter plots.

### Metagenomic functional prediction

The open source software PICRUSt (Phylogenetic Investigation of Communities by Reconstruction of unobserved States; v. 1.0.0-dev) was used to predict the functional capacity of microbiome using 16S rRNA gene sequencing data and Greengenes (v. 13.5) reference database (Langille et al., 2013). To make our open-reference picked OTUs compatible with PICRUSt, all *de-novo* OTUs were removed and only those that had matching Greengenes identifications were retained. The new OTU table was then used to generate metagenomic data after normalizing the data by copy numbers, and to derive relative Kyoto Encyclopedia of Genes and Genomes (KEGG) Pathway abundance (Langille et al., 2013). The KEGG data were analyzed using STAMP (STatistical Analysis of Metage- nomic Profiles; Parks and Beiko, 2010).

### Other statistical analysis

The SAS UNIVARIATE procedure (SAS 9.3) was used to test the normality of residuals for α biodiversity data. Non-normally distributed data were log transformed and then used to assess the effect of sampling using the SAS MIXED procedure. The SAS MIXED procedure was used, as described above, to test for significant changes in the proportions of different phyla among the groups of interest. Differences between groups were considered significant at *P* < 0.05 while trends were observed at *P* < 0.1.

## Results

### Descriptive analysis

Over the 6 days of treatment, daily i.r. infusion of the peptide did not show any effect on the weight, stool consistency, and presence of blood in the feces. The data are consistent with our previous findings (Rabbi et al., 2014; data not shown).

### Sample assessment by illumina sequencing

After sacrifice, we collected fecal and colon samples from a total of 12 mice. Among these, the control group received normal saline i.r and the others received CST i.r. (1.5 mg per kg body weight for 6 days). During the DNA extraction process, one colonic mucosa sample from the saline-treated group was discarded because of poor quality or purity, resulting in a total of 12 useable fecal samples and 11 useable colonic mucosa samples for Illumina sequencing. For fecal samples, a total of 328,085 sequences were generated. After quality-filtering steps, an average of 27,340 high quality sequences per sample was obtained. For colonic mucosa samples, a total of 207,123 sequences were generated. After quality-filtering steps, an average of 18,829 high-quality sequences per sample was obtained.

### CST exposure did not significantly influence α-diversity in fecal and colonic mucosa samples in mice

Bacterial richness and diversity from both fecal and colonic mucosa samples between control and CST-treated groups were calculated. However, no significant differences were observed in both fecal and colon samples (data not shown).

### CST treatment significantly influenced β-diversity in fecal samples but not in colonic mucosa samples in mice

**Figure 1:**
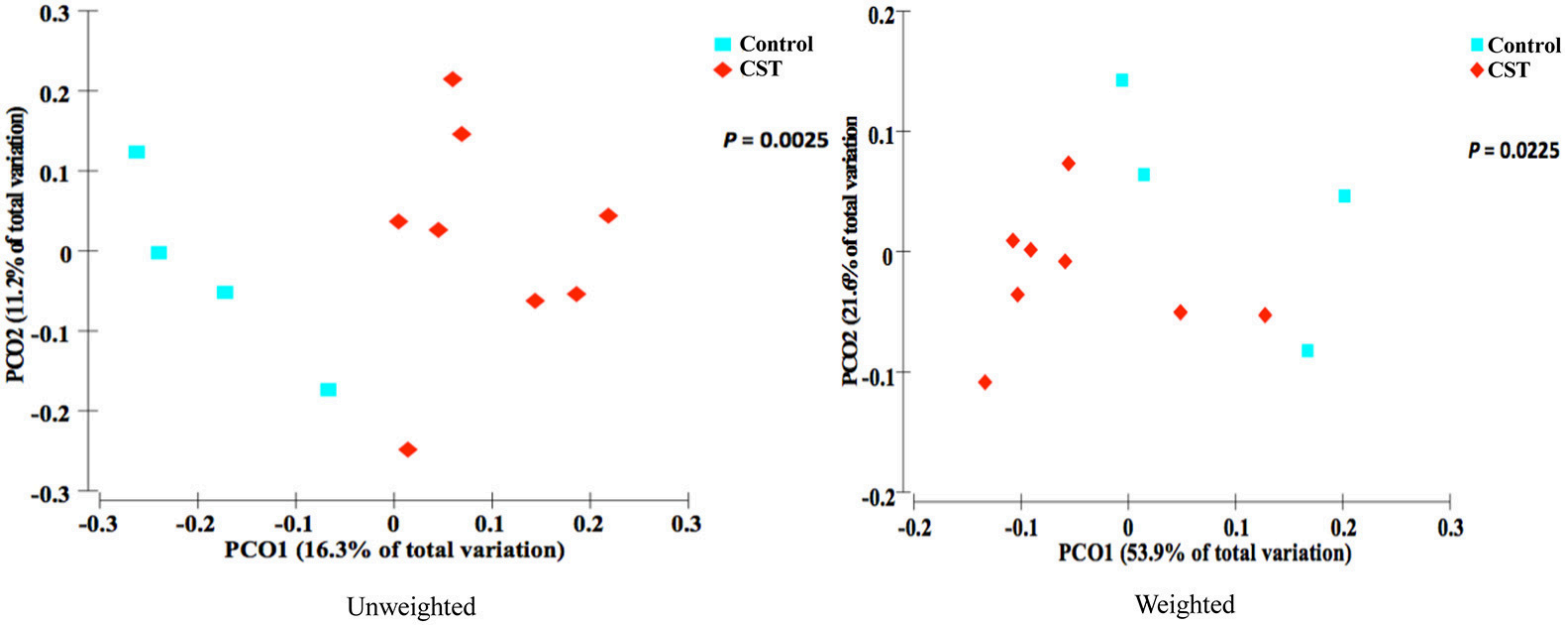
**PCoA based on the unweighted and weighted UniFrac distance metric**. Each colored point represents a fecal sample obtained from one mice and it is colored according to different treatment (CST or Control). *P*-values were calculated using PERMANOVA. Samples clustered according to treatment status of the mice (*P* < 0.05).

**Figure 2:**
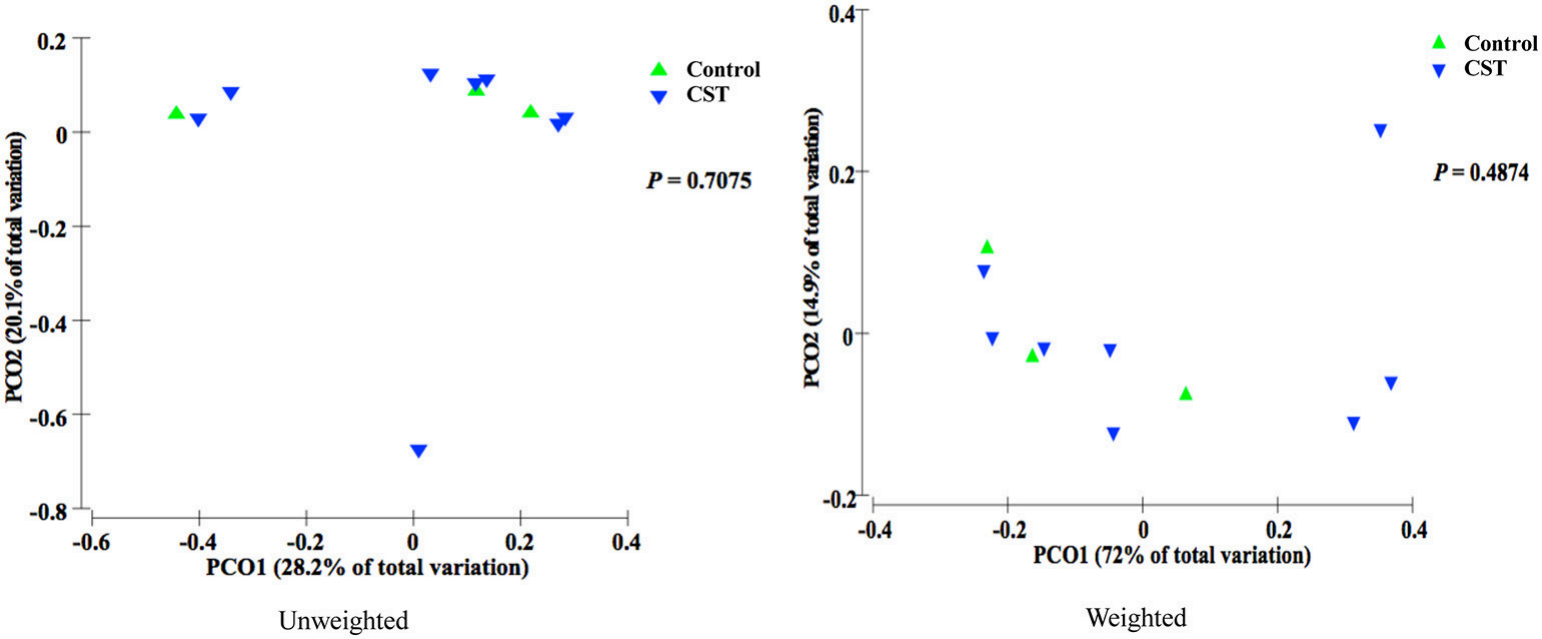
**PCoA based on the unweighted and weighted UniFrac distance metric**. Each colored point represents a colonic mucosa-associated sample obtained from one mice and it is colored according to different treatment (CST or Control). *P*-values were calculated using PERMANOVA. Samples did not cluster according to treatment status of the mice (*P* > 0.05).

Bacterial communities from fecal samples of CST-treated mice clustered separately (*P* < 0.05) from controls suggesting that the treatment modified the fecal bacterial profile (Figure 1). However, there was no significant change in the bacterial community composition in colonic samples in CST-treated mice compared to controls (Figure 2).

### CST treatment influenced fecal but not colonic mucosa- associated bacterial community composition at the phylum level in mice

**Figure 3:**
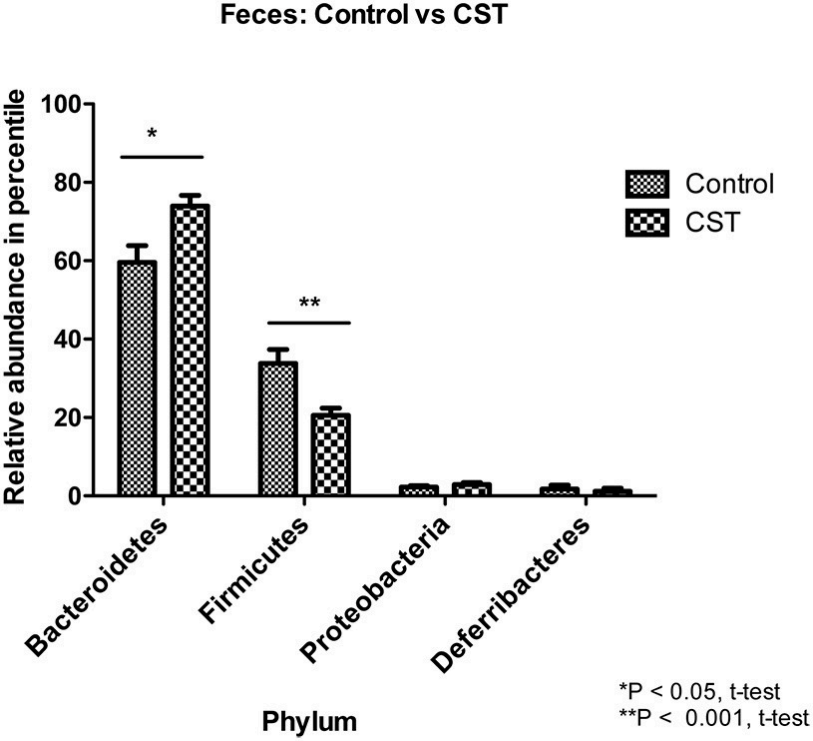
**Effect of CST treatment on the abundant phyla (≥1%) present in the fecal samples**. After quality filtering steps, 10 bacterial phyla were identified in fecal samples. Among these, four phyla were considered abundant within the community (≥1%), including Firmicutes, Bacteroidetes, Proteobacteria, and Deferribacteres. CST treated mice had higher proportion (*P* < 0.05, *t*-test) of Bacteroidetes and lower abundance (*P* < 0.01, *t*-test) of Firmicutes in the feces compared to control animals.

In the fecal samples, a total of 10 phyla were identified, of which four phyla were considered to be abundant within the community (≥1%); these included Firmicutes, Bacteroidetes, Proteobacteria, and Deferribacteres. The other six phyla were in low abundance within the community (<1%) and included Actinobacteria, Cyanobacteria, Fibrobacteres, TM7, Tenericutes, and Verrucomicrobia (Table 1). Among the four abundant phyla, CST treatment increased the relative abundance of Bacteroidetes (*P* < 0.05) and decreased the Firmicutes proportion (*P* < 0.001) in the feces (Figure 3).

**Table 1:** **Relative abundances of bacterial phyla in fecal samples**.

**Phylum**	**Groups**	**Mean percentage of sequence in total bacterial community**	**SEM**
Unclassified	Control	0.496921	0.092571
	CST	0.414249	0.04264
Actinobacteria	Control	0.511464	0.202226
	CST	0.187706	0.09371
Bacteroidetes	Control	59.59139	4.253899
	CST	73.99889	2.680188
Cyanobacteria	Control	0.017379	0.006038
	CST	0.152001	0.041196
Deferribacteres	Control	1.709693	1.021632
	CST	1.143747	0.782717
Fibrobacteres	Control	0.001941	0.00115
	CST	0.000541	0.000541
Firmicutes	Control	33.80289	3.56062
	CST	20.55645	1.827242
Proteobacteria	Control	2.218421	0.409325
	CST	2.872719	0.488761
TM7	Control	0.003883	0.002301
	CST	0.003098	0.001035
Tenericutes	Control	0.314479	0.089961
	CST	0.404554	0.187125
Verrucomicrobia	Control	1.331539	0.730169
	CST	0.266048	0.129101

**Figure 4:**
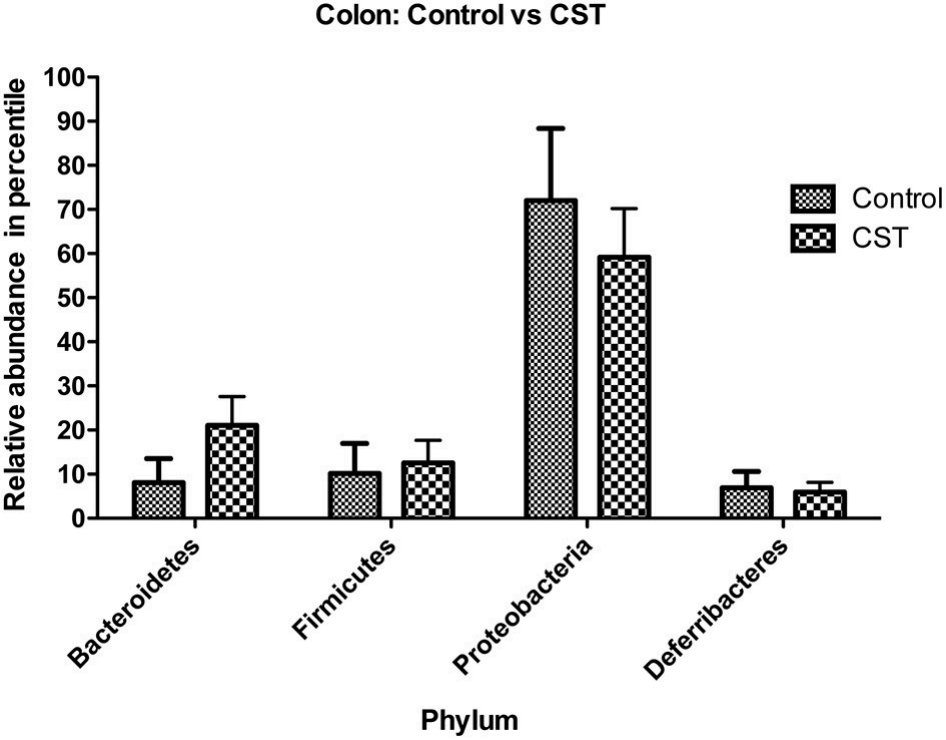
**Effect of CST treatment on the abundant phyla (≥1%) present in the colonic mucosa-associated samples**. After quality filtering steps, 19 bacterial phyla were identified in colonic mucosa samples. Among these, four phyla were considered abundant within the community (≥1%), including Firmicutes, Bacteroidetes, Proteobacteria, and Deferribacteres. CST treatment did not change the abundance of these phyla in colonic mucosa samples.

In the colonic mucosa samples, a total of 19 phyla were identified, of which four phyla were considered to be abundant within the community; these included Firmicutes, Bacteroidetes, Proteobacteria, and Deferribacteres. The other 15 phyla were in low abundance within the community, and included Acidobacteria, Actinobacteria, Armatimonadetes, Chlamydiae, Chlorobi, Cyanobacteria, Fibrobacteres, Lentisphaerae, OD1, OP3, Planctomycetes, Spirochaetes, TM7, Tenericutes, and Verrucomicrobia (Table 2). CST treatment had no significant impact on the relative abundance of bacterial phyla (Figure 4).

**Table 2:** **Relative abundances of bacterial phyla in colonic mucosa samples**.

**Phylum**	**Groups**	**Mean percentage of sequence in total bacterial population**	**SEM**
Unclassified	Control	0.122989	0.050167
	CST	0.291527	0.19307
Acidobacteria	Control	0.025735	0.010402
	CST	0.033388	0.00685
Actinobacteria	Control	0.095085	0.095085
	CST	0.072405	0.031928
Armatimonadetes	Control	0	0
	CST	0.000879	0.000879
Bacteroidetes	Control	8.06612	5.42359
	CST	21.0435	6.556034
Chlamydiae	Control	0	0
	CST	0.00306	0.002007
Chlorobi	Control	0	0
	CST	0.002098	0.002098
Cyanobacteria	Control	0.039247	0.022309
	CST	0.175241	0.063441
Deferribacteres	Control	6.923494	3.656936
	CST	5.949701	2.168418
Fibrobacteres	Control	0	0
	CST	0.013779	0.012973
Firmicutes	Control	10.13424	6.855156
	CST	12.51866	5.161134
Lentisphaerae	Control	0	0
	CST	0.00457	0.00457
OD1	Control	0	0
	CST	0.004988	0.00332
OP3	Control	0	0
	CST	0.001604	0.001604
Planctomycetes	Control	0.002067	0.002067
	CST	0.004664	0.001662
Proteobacteria	Control	72.02106	16.32041
	CST	59.16764	11.01085
Spirochaetes	Control	0	0
	CST	0.082258	0.082258
TM7	Control	0	0
	CST	0.007409	0.00482
Tenericutes	Control	0.037096	0.019253
	CST	0.371448	0.170574
Verrucomicrobia	Control	2.532872	2.52412
	CST	0.142801	0.117295

### CST treatment influenced fecal bacterial community composition at the lower taxonomical levels in mice

**Figure 5:**
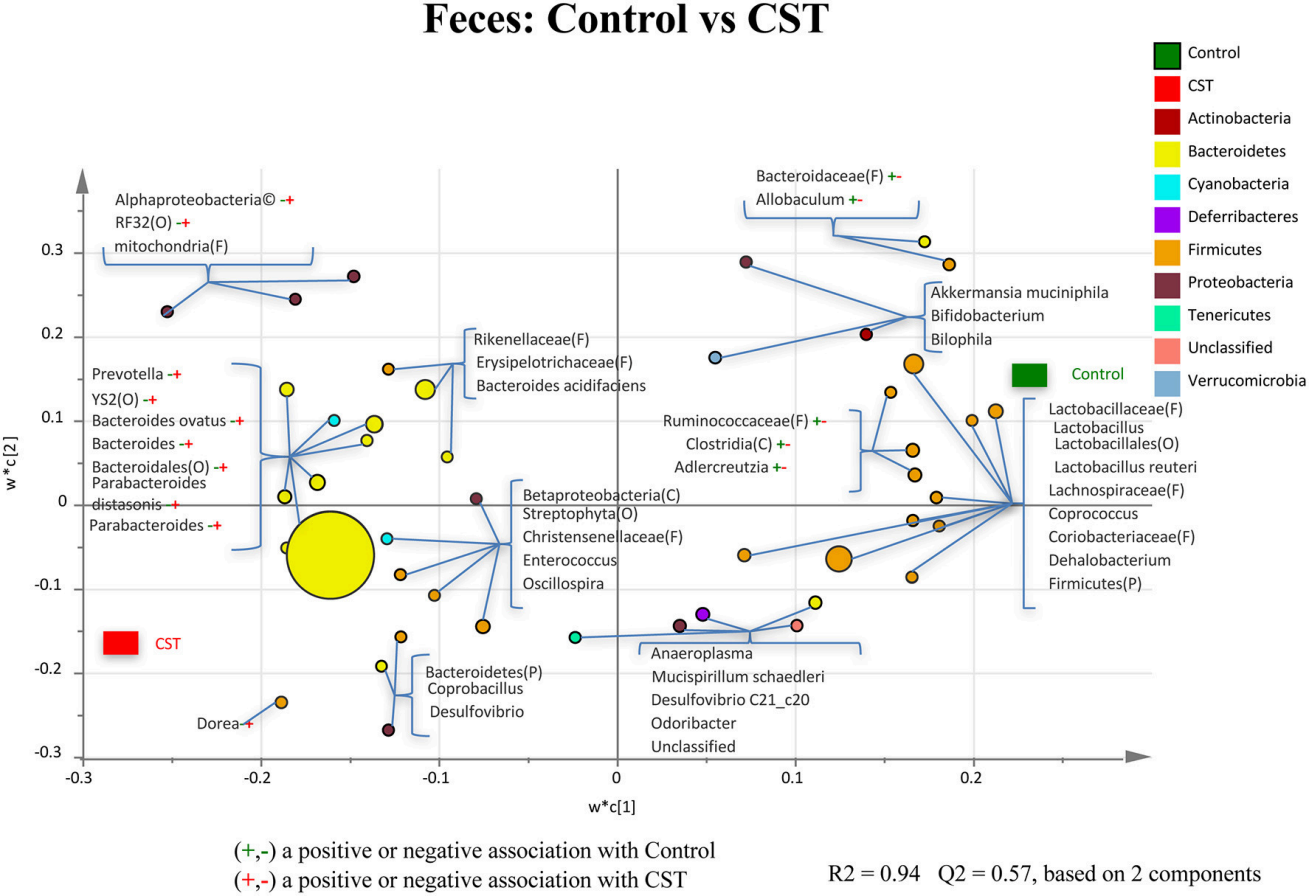
**Partial least square discriminant analysis (PLS-DA) of bacterial communities comparing taxa that were associated with the Control or CST treatments in the mice fecal samples**. All taxa are colored based on the phyla to which they belong. Some sequences could only be affiliated to phylum (P), order (O), family (F), or class (C) levels. Specific taxa were significantly associated with each treatment group, which may be an indicator of an alteration in the physiological or metabolic processes that the taxa may influence.

A total of 86 bacterial taxa were identified. While majority of taxa were classified at the genus or species levels, some were only classified at the phylum (P), class (C), order (O), or family (F) levels. Of the 86 taxa, 54 taxa were considered abundant within the community, while 32 were in low abundance. Results of the relative abundance of various genera with percentages of sequences ≥0.01% of community were analyzed using PLS-DA to identify bacteria that were most characteristic of CST or Control treatments. The PLS-DA analysis showed that the genera *Prevotella, Bacteroides, Ovatus, Parabacteroidesdistarosis, Parabacteroides*, and *Dorea* were positively associated with the CST treatment in the fecal samples (*R*^2^ = 0.94, *Q*^2^ = 0.57; Figure 5). In addition, members of Alpharoteobacteria (Class), Bacteroidales (Order), RF32 (Order), and YS2 (Order) also showed a positive association with CST treatment in the fecal samples (*R*^2^ = 0.94, *Q*^2^ = 0.57). A negative association with the members of *Adlercreutzia, Allobaculum*, Bacteroidaceae (Family), Clostridia (Class) and Ruminococcaceae (Family) were evident in the fecal samples collected from CST-treated mice (*R*^2^ = 0.94, *Q*^2^ = 0.57).

### CST treatment influenced colonic mucosa-associated bacterial community composition at lower taxonomical levels in mice

**Figure 6:**
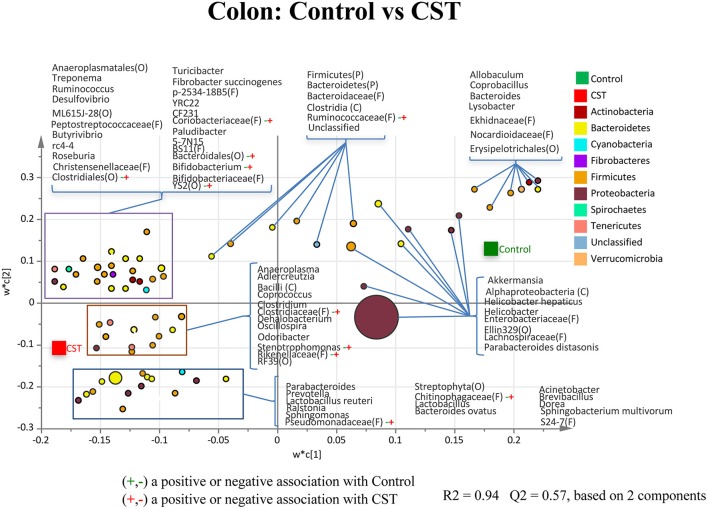
**Partial least square discriminant analysis (PLS-DA) of bacterial communities comparing taxa that were associated with the Control or CST treatments in the mice colonic mucosa-associated samples**. All taxa are colored based on the phyla to which they belong. Some sequences could only be affiliated to phylum (P), order (O), family (F), or class (C) levels. Specific taxa were significantly associated with each treatment group, which may be an indicator of an alteration in the physiological or metabolic processes that the taxa may influence.

A hundred and seventy nine taxa were identified. Of these 84 taxa were considered abundant within the community, while 95 taxa were in low abundance. The relative abundance of various genera/taxa with sequence percentages ≥0.01% of community were analyzed using PLS-DA to identify bacteria that were most characteristic of the CST or control treatments. The PLS-DA analysis of the colonic mucosa samples showed that genera *Bifidobacterium* and *Stenotrophomonas* had a positive association with the CST treatment (*R*^2^ = 0.32, *Q*^2^ = 0.242; Figure 6). Members of Bacteroidales (Order), Chitinophagaceae (Family), Clostridiaceae (Family), Clostridiales (Order), Coriobacteriaceae (Family), Pseudomonadaceae (Family), Rikenellaceae (Family), Ruminococcaceae (Family), and YS2 (Order) also showed a positive association with the CST treatment in the colonic mucosa samples (*R*^2^ = 0.32, *Q*^2^ = 0.242; Figure 6).

### CST treatment significantly influenced the predicted functional and metabolic pathways of fecal and colonic mucosa-associated microbiota in mice

**Figure 7:**
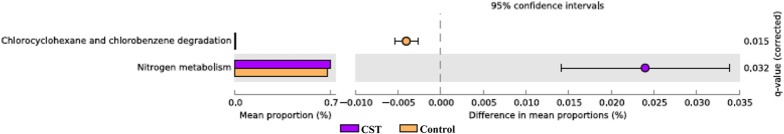
**Subsystems and pathways enriched or decreased within the Catestatin (CST) or (Control) mice fecal samples**. Corrected *P*-values were calculated using the Storey FDR correction. Subsystems or pathways overrepresented in the CST or Control mice fecal samples have a positive or (negative) difference between mean proportions and are indicated by purple or (orange) coloring, respectively.

**Figure 8:**
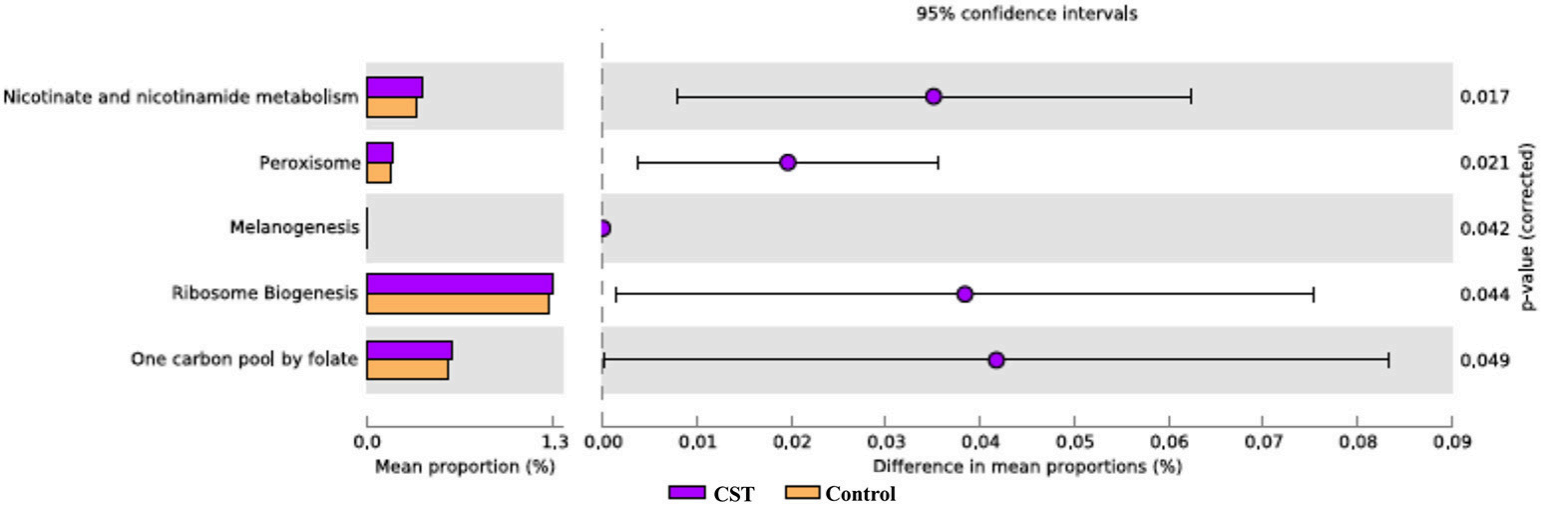
**Subsystems and pathways enriched or decreased within the Catestatin (CST) or (Control) mice colonic mucosa-associated samples**. Subsystems or pathways overrepresented in the CST or Control mice colon samples have a positive or (negative) difference between mean proportions and are indicated by Purple or (orange) coloring, respectively.

To determine the functional KEGG pathways that could be associated with the observed microbial changes, we compared the functional pathways for the microbiota in the fecal and colonic mucosa samples from the CST-treated group with those of the control mice. Several metabolic pathways were determined. Subsystems or pathways that had a significant positive or negative correlation with CST treatment are shown in Figures 7, 8. In the fecal samples from CST-treated mice, chlorocyclohexane and chlorobenzene degradation were underrepresented (*P* = 0.015; Figure 7). However, nitrogen metabolism was enriched in the fecal samples from CST-treated mice (*P* = 0.033; Figure 7). In the colonic mucosa samples from CST treated mice, nicotinate and nicotinamide metabolism, cell division and ribosome biogenesis were enriched compared to the controls (*P* < 0.05; Figure 8).

## Discussion

The mammalian intestine continuously encounters more microorganisms than any other tissue, and survival of the mammals largely depends on their unique adaption in the world of microorganisms. Specific intestinal epithelial cells release several antimicrobial peptides, which are critical for maintaining a stable ecological environment that favors commensal and targeting pathological microorganisms (Bals, 2000). Moreover, these are also important for inhibiting ongoing inflammatory responses. The CST, a highly conserved CgA peptide that is present in intestinal EC cells, has been described as a peptide with some immunomodulatory activities during acute experimental colitis (Rabbi et al., 2014) and restricted *in vitro* antibacterial activities (Aslam et al., 2012), but also antifungal and antiviral activity (Boman et al., 1993; Dorschner et al., 2001). Moreover, in a recent report, it has been observed that the CgA, the precursor of CST strongly regulates human gut microbiome (Zhernakova et al., 2016). However, there are no documented studies demonstrating the effect of CST on gut microbiota using *in vivo* models. Here, we show that i.r. infusion of CST modulates gut microbiota composition under physiological conditions.

Based on the α-diversity, we observed that bacterial richness and diversity in both fecal and colonic mucosa samples did not change after CST administration. However, β-diversity analysis revealed that CST-treated mice had a fecal microbial composition that was different from the control group (*P* < 0.05, both weighted and unweighted) suggesting that a short-term exposure of this peptide in the gut might change the bacterial composition profile. This is in accordance with studies demonstrating the effect of other antibacterial peptides released by Paneth cells (*i.e.*, defensin) on gut microbiota (Salzman et al., 2010).

Recent investigation shows that intestinal inflammatory conditions, such as inflammatory bowel disease (IBD) or inflammatory bowel syndrome (IBS), are associated with altered intestinal homeostasis (Collins, 2014; Comito et al., 2014). Although microbial dysbiosis has been suggested to be a cause of intestinal pathophysiological conditions, this is still controversial, however, gut dysbiosis can take part of the entire process. In parallel, in human and animal models of IBS, it has been observed that microbial diversity is significantly altered (Collins, 2014; Comito et al., 2014). In the context of IBS, although an exact causal microbe has not yet been identified, a reduction in the microbial diversity has been documented and this temporal gut microbiota instability can result in altered host physiology, resulting in heterogeneous symptoms such as those observed in IBS patients (Collins, 2014; Comito et al., 2014). At the phylum level, IBS patients have a relative higher abundance of Firmicutes and lower abundance of Bacteroidetes (Collins, 2014). In our study, we observed that CST treatment is significantly associated with a relative reduction of Firmicutes in the feces compared with saline-treated mice. Conversely, CST treatment was associated with a significant relative increased abundance of Bacteroidetes in the feces compared with saline-treated mice. Beside IBS, also in colitic conditions, studies have demonstrated a relative reduction in Bacteroidetes proportion (Nagalingam et al., 2011). In a recent article, we also observed that acute dextran sulfate sodium induced colitic mice have a lower relative abundance of Bacteroidetes in their fecal samples compared to control (Munyaka et al., 2016). In addition to colonic pathologies, studies demonstrated that Firmicutes are significantly more abundant relative to Bacteroidetes in obese mice compared to lean mice (Kallus and Brandt, 2012); these results were also observed in humans (Kallus and Brandt, 2012). In our study, CST treatment was associated with a significant abundance of Bacteroidetes relative to Firmicutes in fecal samples, which was opposite to results from obese animals and humans. However, these changes in Bacteroidetes and Firmicutes abundance were not observed in the colonic mucosa samples suggesting that prolonged administration of this peptide might be required to observe a possible change in the colonic wall. Overall, this study for the first time showed the *in vivo* effect of CST on murine gut microbiota, which was not predictable from the *in vitro* effect of CST on *S. aureus* and *E. coli* (Boman et al., 1993; Dorschner et al., 2001). As gut microbiota is complex and composed of many bacteria, which might not cultivable yet *in vitro*, their relative abundance *in vivo* can be captured through high-throughput sequencing.

The CST treatment also caused microbial alteration at lower taxonomic levels. We observed that certain bacterial taxa were positively associated with CST treatment in both fecal and colonic mucosa samples. Among these taxa, genera *Bacteroides* and *Parabacteroides* showed a positive association with CST treatment in the fecal samples. Both of these belong to the Bacteroidales order, which also showed a positive association with CST treatment in the colonic mucosa samples. *Bacteroides* and *Parabacteroides* spp. represent ~25% of the colonic microbiota and are commensal to the host when present in the gut (Salyers, 1984). These anaerobic rods can transform simple and complex sugars into volatile fatty acids, which can be absorbed by the large intestine as a nutrient. *Bacteroides thetaiotaomicron* has several starch-binding genes and can produce significant amount of glycosylhydrolases, which can be crucial to prevent obesity (Wexler, 2007). This might explain why *Bacteroidetes* are more abundant in lean mice compared to obese mice. Beside the enormous starch-utilizing capacity, *Bacteroides* spp. are important for developing gut immunity. For example, *B. thetaiotaomicron* can stimulate Paneth cells to produce Paneth cell protein (Ang4), which is lethal to certain pathogenic microorganisms (e.g., *Listeria monocytogenes*; Hooper et al., 2003). In addition, *Bacteroides fragilis* produces zwitter ionic polysaccharide (ZPS), which is important for developing CD4 T cells. ZPS-activated CD4 T cells produce interleukin-10 (IL-10), which is essential to prevent abscess formation and other unchecked inflammatory responses (Mazmanian and Kasper, 2006; Wexler, 2007; Round and Mazmanian, 2010). Increased *Bacteroides* abundance in mice in response to CST exposure might be beneficial to control obesity and inflammatory conditions such as IBD or IBS. These results might also explain the underlying mechanisms for improving gut inflammation that we observed previously in colitic mice exposed to CST (Rabbi et al., 2014).

Finally, our metagenomic prediction analysis helped to understand the functionaly of microbiota in the given environment (Chistoserdovai, 2010). Using this approach, we observed that certain subsystems or pathways were enriched after CST treatment suggesting that, although, we were not able to see any effect for some markers studied after 6 days, treatment with CST modified specific functional activities of the microbiota. In this context, CST treatment induced functional alteration in the murine intestinal microbiota, with some metabolic pathways enriched in the mucosal microbiota of fecal and colonic mucosa samples compared to the control mice. However, since this was prediction and the specific changes observed might not directly influence the host's metabolic capacity, further studies might shed more light on this topic. In conclusion, our findings provide new insight into gut microbiota modulation by CST. We observed an alteration in the microbial profile in response to CST treatment, which was more prominent in the feces than in colonic mucosa-associated bacterial community. This is an observation based on a small number of samples, and the result of this study needs to be further validated in a larger experiment associated to a paired analysis of feces. Our results suggest new avenues for the development of a potential new anti-microbial peptide, which could be used as a therapeutic agent to treat several gastrointestinal conditions such as IBD, IBS, however, further studies are warrant.

## Author contributions

JEG, MM conceived and designed the study. MM provided the peptide. MFR, PM, NE conducted the experiment and performed the analyses. MFR, EK, PM, NE, and JEG interpreted the data and wrote the manuscript.

## Funding

This study was supported by grants from Research Manitoba, the Canada Foundation for Innovation, the Children's Hospital Research Institute, the Canadian Institutes of Health Research of Manitoba, Natural Sciences and Engineering Research Council of Canada and Crohn's and Colitis Canada to JEG. Manitoba Health Research Council, Mitacs, Crohn's, and Colitis Canada and the Children's Hospital Research Institute supported MFR and NE through student fellowships.

### Conflict of interest statement

The authors declare that the research was conducted in the absence of any commercial or financial relationships that could be construed as a potential conflict of interest.
